# Extraction of Chitin from Black Soldier Fly (*Hermetia illucens*) and Its Puparium by Using Biological Treatment

**DOI:** 10.3390/life13071424

**Published:** 2023-06-21

**Authors:** Anqi Xiong, Linsen Ruan, Kaiyu Ye, Zhiyong Huang, Chan Yu

**Affiliations:** 1 State Key Laboratory of Biocatalysis and Enzyme Engineering, School of Life Sciences, Hubei University, Wuhan 430062, China; xaq208193369@163.com (A.X.); ruanlinsen1997@gmail.com (L.R.); 202121107012381@stu.hubu.edu.cn (K.Y.); 2 Chinese Academy of Sciences, National Technology Innovation Center of Synthetic Biology, Tianjin Institute of Industrial Biotechnology, Tianjin 300308, China

**Keywords:** *Hermetia illucens*, chitin, biological approach, FTIR

## Abstract

Chitin is the second-largest natural polymer polysaccharide in nature. Due to its important physical and chemical properties and excellent biocompatibility, safety, and biodegradability, it is widely used in agriculture, medicine, food, environmental protection, and other fields. However, traditional extraction methods cause environmental pollution and damage the structure of chitin. Bioprocessing is an emerging technology that shows great potential. In this research, the puparia and adults of black soldier fly (BSF) (*Hermetia illucens* L.) were used as raw materials. A continuous fermentation method was designed to extract chitin, by using *Bacillus subtilis* S4 and *Acetobacter pasteurianus* AS1.41. The Fourier transform infrared spectroscopy identification results showed that the extracted sample was α-chitin. Under continuous fermentation conditions, the deproteinization (DP) rate, demineralization (DM) rate, chitin yield (CY), and deacetylation degree (DD) of puparium chitin were 33.33%, 94.92%, 59.90%, and 18.52%, respectively. Meanwhile, the DP rate, DM rate, CY, and DD of adult chitin were 46.63%, 90.93%, 47.31%, and 37.38%, respectively. For BSF, *B. subtilis* S4 had a certain DP ability, and *A. pasteurianus* AS1.41 had a good DM effect. Moreover, BSF at different developmental stages could affect CY, and a higher concentration of NaOH was more favorable for deacetylation. Overall, simultaneous continuous fermentation could be a new biological approach to extract chitin from BSF.

## 1. Introduction

Chitin is a linear amino polysaccharide polymerized from β-1,4-N-acetyl-D-glucosamine and also is the second-most-abundant natural polymer polysaccharide in nature, second only to cellulose, widely found in the exoskeletons of crustaceans and insects and the cell walls of some fungi and algae; it is known as “the sixth element of human life” [1,2]. Chitin possesses a rigid structure and is not soluble in water, dilute acid, dilute alkali, ethanol, or other organic solvents. However, it can undergo deacetylation in alkaline conditions to yield chitosan. Chitin and chitosan have unique functional properties such as biocompatibility, biodegradability, and no toxic side effects. In addition, chitosan also has good antibacterial and anti-oxidation capabilities and excellent air permeability and is often used in the medical field to prevent wound infection and to build drug delivery systems. Chitin is also widely used in many areas, such as wastewater treatment, heavy-metal recovery, cosmetics, agriculture, and medical care [3,4]. The chitin market is estimated to reach USD 2.941 billion in 2027 [5]. Currently, commercially available chitin is extracted from the waste of crustaceans, such as shrimp and crab shells [6]. However, the content of chitin and its physicochemical properties vary among crustacean species, and such variations in raw materials are generally undesirable for industry [7,8].

Besides crustaceans and fungi, insects are another promising and sustainable source of chitin and chitosan. Compared to crustaceans, insects are not subject to seasonality and reproduce easily due to their high reproductive rate. Notably, as biotransformers, insects can be developed as effective alternatives to crustaceans as sources of chitin and chitosan for greater ecological and economic sustainability. Depending on the species, the chitin content in molts of insects can be as high as 35% by dry weight [9]. It is well known that flies are biodegradable organic waste species, especially black soldier fly. Black soldier fly (BSF) (*Hermetia illucens* L.) is an ideal eco-friendly insect, native to North America, which feeds on animal manure and domestic waste and converts biomass nutrients into biomass, which can effectively reduce pollution and produce highly valuable animal protein feed [10]. It is widely used because of its fast reproduction, wide range of recipes, large food intake, low nutritional requirements, high absorption conversion rate, and high safety. However, due to the short life cycle of BSF, a large amount of biological wastes, namely, puparia and dead adults, are produced every year. If they are not handled properly, they may cause environmental pollution [11]. Waste from this process can be a year-round source of free chitin. It has been reported that insect exoskeletons are rich in chitin, and the exoskeleton of black soldier fly contains up to 35% chitin [9]. Therefore, useful biopolymers such as chitin and chitosan could be obtained from BSF waste, and the environmental pollution problem could be alleviated.

The extraction of chitin is usually divided into three stages, deproteinization (DP), demineralization (DM), and decolorization (DC), and DP and DM are mainly achieved by chemical or biological methods, such as the chemical acid–base method, enzymatic method, microbial fermentation method, EDTA method, DES method, etc. [12,13,14]. Decolorization or color bleaching is an optional step, though almost no researcher skips this step due to certain factors. Exoskeletons or crustaceans naturally have pigments that give each a different color [15]. Decolorization is performed using several methods: potassium permanganate, hydrogen peroxide (H_2_O_2_), or sodium hypochlorite (NaClO). Today, industrially, chitin is chemically purified from marine crustaceans. However, the chemical extraction process may destroy the chitin structure, resulting in an unstable quality of the extracted chitin [16]. In addition, the large amount of waste liquid produced by the acid–base process will cause serious environmental pollution, and the subsequent waste liquid treatment process is expensive, which is contrary to the development trend of green, healthy, and environmental protection. The biological method of chitin extraction has gained increased interest in recent years. Among many biological methods, microbial fermentation technology has attracted researchers for its lower cost than enzymatic methods. By contrast, it has the characteristics of mild working conditions, low cost, simple operation, and environmental friendliness [17,18,19]. In the process of extracting biological chitin, enzyme-producing strains and acid-producing strains are usually used for DP and DM. The DP of microorganisms is mild, which is beneficial to the retention of some active substances to obtain functional chitin with more antibacterial and anti-oxidative properties. This helps to address some of the limitations of traditional methods. However, there are often competitive inhibition and interference between enzyme-producing microorganisms and acid-producing microorganisms, which makes the DP and DM operations very complicated, such as repeated sterilization and collection of residues. During the co-cultivation process, *Acetobacter pasteurianus* uses ethanol as a carbon source, converts it into acetic acid, and can inhibit or kill part of *Bacillus subtilis*, so that there is little interference between the two different strains. Therefore, the co-culture of Bacillus subtilis and Pasteurella to extract chitin is feasible and has the advantages of no need to sterilize, no need to replace the medium, and a short fermentation time. Therefore, the continuous fermentation method is a new strategy and a competitive method for chitin extraction.

In this study, the puparia and adults of BSF were used as raw materials to extract chitin by the co-culture of *Bacillus subtilis* and *Acetobacter pasteurianus*. Compared with the chemical method, the extraction efficiency, DP rate, DM rate, and DD of the two methods were analyzed; the advantages and disadvantages of the extraction effect were compared. This process provides a new, sustainable source for commercial chitin.

## 2. Materials and Methods

### 2.1. Substrate and Bacterial Strains

**Figure 1:**
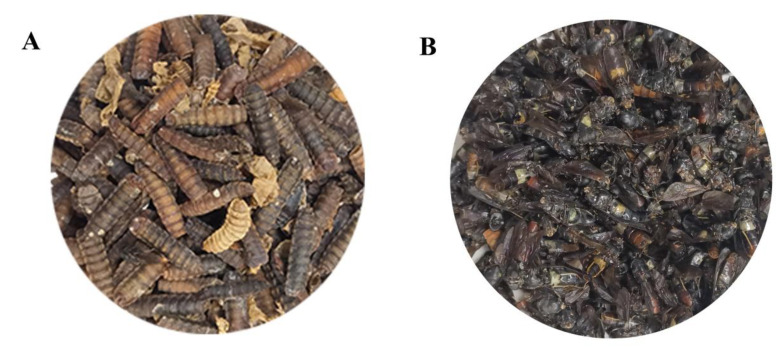
Puparia (**A**) and adult (**B**) black soldier fly (BSF).

Black soldier fly was obtained from a colony maintained throughout the year in Huazhong Agricultural University, Wuhan, China (Figure 1). The breeding systems were in a greenhouse at 27.5 °C with 70% relative humidity. The larvae were fed with an artificial diet of wheat bran and wheat flour (1:1, Wuhan City, China). Exfoliated puparia and naturally dead adults were collected (Figure 1), dried in an oven at 60 °C, and then, pulverized into a powder and stored at −20 °C.

*Acetobacter pasteurianus* AS1.41 was purchased from Guangdong Microbial Culture Collection Center, China. It was inoculated into a medium containing 10 g⋅L^−1^ of yeast extracts, 10 g⋅L^−1^ of glucose, and 2% (*v*/*v*) ethanol and then cultured at 30 °C for 220 rpm. The final bacterial liquid yielded a concentration of approximately 10^8^ CFU⋅mL^−1^ before use.

*Bacillus subtilis* S4 was maintained at Huazhong Agriculture University, Wuhan, China. It was activated and proliferated in a solid nutrient medium containing 10 g⋅L^−1^ of yeast extract, 5 g⋅L^−1^ of tryptone, and 10 g⋅L^−1^ of NaCl. The liquid culture was obtained by shaking with the same nutrient broth at 30 °C for 220 rpm until the bacterial concentration reached approximately 10^8^ CFU⋅mL^−1^.

### 2.2. Chemical Extraction

In accordance with Fadlaoui et al. [20], the sample was demineralized and deproteinized by a dilute acid and alkaline solution. In particular, 2.5 g of the sample was accurately weighed and placed in 25 mL of 5% HCl, soaked at room temperature until no bubbles were generated, washed until the filtrate was neutral, and dried in the oven at 60 °C for 6 h. Then, the sample was deproteinized with 2% NaOH solution at 70 °C–90 °C for 2 h, washed until the filtrate was neutral, and dried in the oven at 60 °C for 6 h.

### 2.3. Biological Extraction

**Figure 2:**
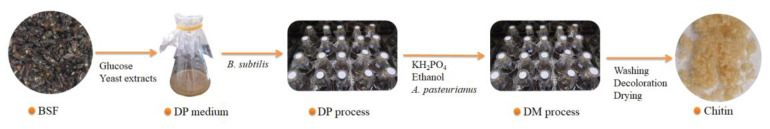
Schematic of biological extraction of chitin from *B. subtilis* and *A. pasteurianus*.

*B. subtilis* and *A. pasteurianus* were used to extract chitin by continuous co-fermentation to remove proteins and minerals from pupae and adults, respectively (Figure 2). In particular, an inoculum of 3% (*v*/*v*) *B. subtilis* was transformed into 50 mL of fermentation medium composed of 50 g⋅L^−1^ of BSF, 50 g⋅L^−1^ of glucose, and 1 g⋅L^−1^ of yeast extracts in a 250 mL flask. The fermentation medium was cultured in a shaker at 30 °C and 220 rpm for 3 days. Afterwards, 6% of ethanol (*v*/*v*) and 5 g⋅L^−1^ of KH_2_PO_4_ were fed into the fermentation liquid, and then, a 5% (*v*/*v*) seed culture of *A. pasteurianus* was inoculated. The DM process was performed for 2 days at 30 °C and 220 rpm. The fermentation residuals were collected with a nylon cloth, washed, and dried at 105 °C.

### 2.4. Chitin Decolorization and Deacetylation

The DC step aimed at removing the leftover pigments associated with BSF. The dried sample was decolorized with hydrogen peroxide (30%, *v*/*v*) at a ratio of 1:20 (*w*:*v*) at 90 °C for 45 min. The sample was rinsed to a neutral pH and, finally, dried at 60 °C for 24 h in the oven. With this, the chitin extraction process was completed. Deacetylation is the process of removing the acetyl group of the chitin structure. The dried chitin samples were deacetylated with NaOH (30%, *v*/*v*) at a ratio of 1:50 (*w*:*v*) for 3 h at 90 °C. The residue was collected with a nylon cloth and washed to a neutral pH. The wet samples were dried at 60 °C for 12 h [21,22].

### 2.5. Atomic Absorption Measurement

#### 2.5.1. Determination of Protein Content

The nitrogen concentrations in the samples were determined in accordance with the standard method specified by the National Standardization Administration of the People’s Republic of China (NY 525-2020). Under the condition of alkaline medium at 120 °C–124 °C, potassium persulfate was used as the oxidant to oxidize the organic nitrogen compounds to nitrate. Then, the absorbance was measured at wavelengths of 220 and 275 nm using a UV spectrophotometer, and the absorbance of the ammonia nitrate was calculated using the following formula: A = A_220_ − 2A_275_. Thus, the content of total nitrogen was calculated. The DP rate was calculated using Equation (1).

(1) $$DP\left(\%\right)=\left(1-\frac{M_{1}\times {6.25N}_{1}}{M_{0}\times 6.25N_{0}}\right)\times 100,$$

where M_0_ and M_1_ are the weight of the original sample and the residue (g), respectively; 6.25 is the protein coefficient; and N_0_ and N_1_ are the nitrogen contents in the original sample and the residue, respectively.

#### 2.5.2. Determination of Ash Content

The ash content was determined by the weighing method. After the sample was weighed, it was heated at low heat to fully carbonize it until no smoke was present. Then, it was placed in a muffle furnace, burned at 550 °C ± 25 °C for 4 h, cooled to about 200 °C, taken out, and placed in a desiccator to cool for 30 min. Burning was repeated until a constant weight was achieved, and the ash content was calculated. The DM rate was calculated using Equation (2).

(2) $$DM\left(\%\right)=\left(1-\frac{M_{1}\times A_{1}}{M_{0}\times A_{0}}\right)\times 100,$$

where M_0_ and M_1_ are the weight of the original sample and the residue (g), respectively; 6.25 is the protein coefficient; and A_0_ and A_1_ are the percentage of ash in the original sample and the residue (%), respectively.

#### 2.5.3. Determination of Deacetylation Degree

The DD of chitin was determined by acid–base titration. A quantitative amount of the chitosan sample was accurately weighed, dried in a drying oven to a constant weight, placed in a 250 mL conical flask, added with 30 mL of 0.1 mol/L HCl standard solution, and stirred at 20 °C–25 °C until it was completely dissolved. Then, 2–3 drops of the methyl orange indicator (1%) were added, and the free hydrochloric acid was titrated with a 0.1 mol/L of NaOH standard solution. The process was repeated three times, and the DD was calculated using Equation (3).

(3) $$DD(\%)=\frac{(C_{1}V_{1}-C_{2}V_{2})\times 0.016}{G\times 0.0994}\times 100,$$

where C_1_ is the concentration of the HCl standard solution (mol/L), C_2_ is the concentration of the NaOH standard solution (mol/L), V_1_ is the volume of the HCl standard solution (mL), V_2_ is the volume of the consumed NaOH standard solution (mL), G is the sample mass (g), 0.016 is the amount of amino groups equivalent to 1 mL of 1 mol/L of hydrochloric acid (g), and 0.0994 is the theoretical amino group content.

#### 2.5.4. Fourier Transform Infrared Spectroscopy

Infrared analysis of dried samples was performed using an IRTracer-100 FTIR spectrometer. The sample was mixed with potassium bromide at a ratio of 1:100, placed in an agate mortar, ground into a very fine powder, pressed into flakes, and scanned with an infrared spectrometer. The experimental parameters were as follows: wavelength scanning range of 4000–400 cm^−1^, spectral resolution of 4 cm^−1^, and scanning times of 40.

### 2.6. Statistical Analysis

All experiments were performed in triplicate, and the data are expressed as the mean ± the standard deviation (SD). The difference was evaluated by ANOVA on the basis of Duncan’s multiple range test via SPSS Version 25.0 (IBM, Armonk, NY, USA). A *p*-value below 0.05 was considered as a significant difference.

## 3. Results

### 3.1. Deproteinization

Chitin is always associated with proteins and forms a chitin–protein complex [23]. DP is the process of removing the protein associated with chitin, forming a water-soluble amino acid. Deproteination not only removes proteins, but also pigments such as carotenoids and lipids. This process is very important for the medical and biomedical applications of chitin and its derivative, chitosan. In this study, the ability of *Bacillus subtilis* S4 to deproteinize pupae shells and adults of BSF was analyzed by testing the DP rates of biological and chemical methods. 

Table 1 lists the DP rate, DM rate, yield, and DD of chitin. The results showed that the DP rates of pupae and adults were 33.33% and 46.63% after being treated with *Bacillus subtilis* S4. The DP rates of pupae and adults were found to be 75.36% and 89.11%, respectively, after chemical reagent treatment.

### 3.2. Demineralization

In arthropods, chitin together with various proteins and calcium carbonate attached to the protein form a complex network system [24]. Demineralization refers to the removal of mineral components, mainly calcium carbonates or phosphates, from organic matter by releasing CO_2_. Therefore, DM is one of the key steps to remove CaCO_3_ and obtain pure chitin. The higher the acid concentration, the more efficient the solubilization of CaCO_3_ is and the lower the residual ash content in the treated mass. In the present study, *A. pasteurianus* was used to ferment and demineralize BSF. The DM rates of chemically treated puparia and adults were 93.86% and 92.81%, respectively, and those of biologically treated puparia and adults were 95.01% and 90.97%, respectively (Table 1). The results showed that there was no significant difference in the DM rate between the chemical method and the biological method, and the DM rate was above 90%. The demineralization effect of the organic acid produced by *A. pasteurianus* was comparable to that of hydrochloric acid.

### 3.3. Chitin Yield

**Figure 3:**
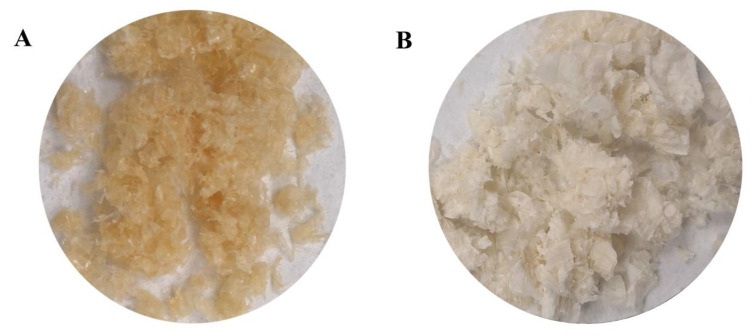
Experimental preparation of chitin (**A**) and chitosan (**B**).

Yield is one of the key indicators for extracting chitin from insects [14]. The economic viability and commercialization of chitin as a product also depend on the yield of samples [25]. After BSF passed through DP and DM, it was decolorized with H_2_O_2_ to obtain chitin (Figure 3A). As shown in Table 1, the chitin yields of biological treatment were 59.90% and 47.31%, whereas those of chemical treatment were 23.82% and 11.99%. The yield of chitin by the biological method was significantly higher than that by the chemical method (*p* < 0.05). The yield of chitin in puparia was significantly higher than that in adults (*p* < 0.05).

### 3.4. Deacetylation

Deacetylation refers to the process of removing the acetamide functional group (NHCOCH_3_) to yield an amine group (–NH_2_) from the chitin backbone by hydrolysis [9]. For insects, the chemical method of deacetylation uses alkali–NaOH to deacetylate chitin [25,26]. The DD is the fraction of the deacetylated units in a chitosan chain, and it determines the percent of free amino groups in the chitosan molecule [14]. Furthermore, The DD affects the physicochemical properties, biodegradability, and immunological activities of chitosan. Thus, it is an important property in chitosan synthesis. The experimental preparation of chitin using NaOH deacetylation at high temperature allowed obtaining chitosan (Figure 3B). The DD measured by the acid–base titration after deacetylation is shown in Table 1. The results showed that, in the same material, no significant difference was found in the DD of chitin prepared by the two methods. The degrees of chitin deacetylation of pupae and adults extracted by the biological method were 18.52% and 37.38%, respectively, and those extracted by the chemical method were 25.72% and 60.23%, respectively. This finding showed that 30% NaOH had a certain deacetylation effect on chitin. 

### 3.5. FTIR Analysis

**Figure 4:**
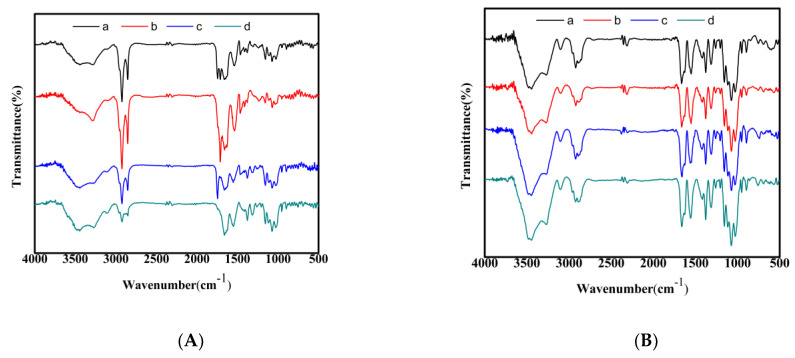
FTIR spectra of (**A**) chitin and (**B**) chitosan. (a) Biological extraction of puparia; (b) biological extraction of adults; (c) chemical extraction of puparia; (d) chemical extraction of adults.

The chitin samples prepared by different treatments were scanned by infrared spectroscopy, and the results are shown in Figure 4A. All four samples had absorption peaks formed by the amide I band (1652 cm^−1^) and amide II band (1555 cm^−1^). A comparison of Figure 4A (a–d) showed that all four chitins had an amide I band absorption peak near 1662 and 1626 cm^−1^, corresponding to C=O amide stretching. For α-chitin, the amide I band had two bands around 1656 and 1621 cm^−1^, whereas β-chitin had only one band at 1655–1660 cm^−1^. Therefore, the four chitins were all α-chitin. 

In Figure 4A (c,d) (chemically prepared puparium and adult chitin), the absorption peaks of the amide II band appeared near 1552 and 1550 cm^−1^. A notable detail is that the absorption peaks of the amide II band appeared near 1537 and 1535 cm^−1^, and the absorption peaks shifted to low wavenumbers, as shown in Figure 4A (a,b) (biological preparation of puparium and adult chitin). This finding showed that the chemically prepared puparium and adult chitin had strong intermolecular hydrogen bonds, whereas the biologically prepared ones had few, weak, and non-obvious hydrogen bonds. The absorption peak around 1540 cm^−1^ can be observed in Figure 4A (biologically and chemically prepared chitin), where proteins normally give rise to absorption [27]. The absorption peaks near 1540 cm^−1^ in Figure 4A (a,b) were larger, indicating more proteins. On the contrary, the absorption peaks near 1540 cm^−1^ in Figure 4A (c,d) were smaller, indicating fewer proteins. However, the biologically and chemically prepared chitin in Figure 4A did not have apparent signals at 1428 and 877 cm^−1^, which corresponded to the characteristic peaks of the carbonate group [28], indicating that the minerals in the chitin were relatively few. This figure also corresponds to the results in Table 1.

Figure 4B reflects the infrared spectroscopic results of chitosan. The FTIR spectra of the four samples showed similar bands, but certain differences could be found in the wavelength and adsorption intensity. The peaks near 3444 and 3271 cm^−1^ could be attributed to the –OH stretching vibration absorption peaks and the –NH stretching vibration absorption peaks, respectively. Two stretching vibration absorption peaks of CH could be found at 2920 and 2889 cm^−1^, respectively. A strong amide I band absorption peak was also observed at 1660–1664 cm^−1^. In addition, 1550–1560 cm^−1^ was found to be the absorption peak of the amide II band, and the position of this peak was different among the four samples. Among them, the peak position of the chemically extracted adult chitosan was the highest. Furthermore, the assignment of bands at 1379 (CH bend), 1417 (C–H bending), 1313 (amide III band, C–N stretching vibration), 1157 (asymmetric bridge C–O–C stretch), and 1028 cm^−1^ (C–O asymmetric stretch of the glycoside ring) were noted, and 894 cm^−1^ was assigned to C–H out-of-plane vibration [21,29]. The free N–H and O–H stretching vibrations were found in the ranges of 3400–3440 and 3500 cm^−1^ and above, respectively. When these functional groups (N–H and O–H groups) were involved in intermolecular hydrogen bonding, their position tended to shift to lower frequencies [30]. In the present study, the absorption peaks around 3400 and 3500 cm^−1^ in the spectra of chitosan indicated the free O–H and N–H groups available. 

## 4. Discussion

Chitin is embedded in the protein matrix in the form of ordered microfibrils to form chitin–protein complexes. Therefore, removing protein is one of the key steps to obtain pure chitin. Comparing the DP efficiency of *B. subtilis* and NaOH on BSF, it can be seen that the strain showed a certain degree of DP ability, but it was not ideal. Improper fermentation conditions during deproteinization of the strain may be the reason for this problem. It has been reported that many factors such as carbon and glucose concentration, inoculum size, temperature, and fermentation time can affect the fermentation process and, thus, the deproteinization efficiency [31,32]. Olfa et al. [33] found that, for deproteinization, sample concentration, temperature, and incubation time had the most-significant effects. Xie et al. [34] used *Exiguobacterium profundum* and *Lactobacillus acidophilus* to extract chitin with a DP rate of 85.9% from shrimp shells. Liu et al. [35] extracted chitin from shrimp shells through a continuous two-step fermentation method, and the DP efficiency of *Lactobacillus rhamnoides* was 96.8%. Li et al. [36] used shrimp by-products as raw materials, inoculated Lactobacillus fermuntum for one-step fermentation, and obtained a chitin DP rate of 85.11%. Kyung-Taek et al. [37] used *Pseudomonas aeruginosa* F722 to extract chitin with DP rates of 63%. This indicated that different strains had different deproteinization abilities. Therefore, future experiments can focus on further optimizing the DP conditions (sample concentration, temperature, fermentation time, inoculum size), exploring other strains with better DP effects.

Generally, the demineralization process is carried out by inorganic or organic acids, such as hydrochloric acid, sulfuric acid, acetic acid, formic acid, lactic acid, etc., among which the most-commonly used reagent is hydrochloric acid. Because of its adverse effects on the environment and the structure and chemical composition of chitin [9], hydrochloric acid has been replaced by organic acids in many cases [15]. In some studies of fermentative extraction of chitin, the DM microorganisms were generally lactic acid bacteria such as *Lactobacillus plantarum* [38], *L. acidophilus* [39], and *Bifidobacterium lactis* [40]. However, acetic acid bacteria have advantages in the successive co-fermentation with protease-producing bacteria. Their carbon source (ethanol) could inhibit the further growth of protease-producing bacteria and reduce their negative impact on the following DM process. In general, *Acetobacter*’s tolerance to alcohol was less than 8% [41]. In the DM process, we tried a 5% *Acetobacter pasteurianus* and 6% ethanol fermentation to remove the minerals of black soldier fly, and the effect was remarkable: the DM rate of the samples reached more than 90%. Therefore, organic acids produced by fermentation are important for efficient demineralization. Oh et al. [37] found the highest correlation between DM and the amount of glucose to be r^2^ = 0.821, respectively. Similarly, Olfa et al. [31] found that glucose concentration had the greatest effect on demineralization, followed by sample concentration, temperature, incubation time, and inoculum size, while the initial pH had little effect. This showed that the efficiency of DM was highly dependent on the glucose concentration. In addition to glucose, a small amount of yeast extract and an appropriate concentration of phosphate were also beneficial to the proliferation of microorganisms and the improvement of enzyme activity [42,43].

Insect chitin production is a key factor for chitin as an economically viable and commercial product. Whole insects generally contain 30–60% protein, 10–25% lipids, 5–25% chitin, 5–10% catechol, and 2–10% minerals (this value varies with species and different stages of development) [9]. Ruben Smets et al. [7] found that the pupae of black soldier fly contain 31.27% proteins, 39.85% lipids, 9.24% ash, and 6.31% chitin. The main minerals are calcium (4.404 g/100 g), potassium (0.611 g/100 g), phosphorus (0.631 g/100 g), magnesium (0.365 g/100 g), and sodium (0.172 g/100 g), and the micro-minerals include manganese (37.6 mg/100 g), iron (6.5 mg/100 g), zinc (6.9 mg/100 g), and copper (1.1 mg/100 g). The exoskeleton of black soldier fly larvae contains up to 35% chitin, contained in the precuticle, the innermost layer of the cuticle [9]. In order to extract chitin from the stratum corneum, a purification process removes the proteins, minerals, pigments, etc., contained in it. PM is an acellular chitin-containing enclosure, which functions to protect the insect against pathogens and abrasion [44]. The lower chitin production in adult BSF than in puparia may be due to the molting process and the inability to feed because of the lack of functional mouthparts, thus affecting PM formation [25]. BSF is widely used in animal feed due to its high crude protein content; however, the chitin contained in it may impair the digestibility of nutrients. Eggink Kylian Manon et al. [45] studied the effect of black soldier fly larva meal (BSFLM) with different chitin content on fish nutrient digestibility and found that Nile tilapia (Oreochromis niloticus) and rainbow trout (Oncorhynchus mykiss) can digest chitin, but their digestibility varies with increasing dietary chitin content, confirming that chitin can serve as a nutrient source and antinutrient for Nile tilapia and rainbow trout.

There is a huge amount of chitosan polysaccharides in nature, which play an important role in the fields of food, medicine, agriculture, environmental protection, and so on. The degree of deacetylation is one of the important indexes to evaluate its performance. The samples treated with 30% NaOH in this study all had lower degrees of deacetylation; however, ideally at least 55% of the N-acetyl groups need to be removed. Currently, several critical factors that affect the extent of deacetylation have been identified, including temperature, time of deacetylation, alkali concentration, and prior treatment applied to the chitin [46]. Qiang Luo et al. [47] treated grasshopper with 60% NaOH, and the DD reached 89.7%. Uche et al. [25] showed that 70% NaOH is the optimal concentration for deacetylation of BSF chitin samples. These findings indicated that the low concentration of NaOH used in the present study may lead to insignificant deacetylation of chitin. In the same material, the DD of biologically treated samples was lower than that of chemically treated samples, maybe due to problems with the prior treatment applied to the chitin, that is too much protein residue in the biologically treated samples. In the same treatment, the DD of puparia was lower than that of adults, maybe because the raw materials of different developmental stages contained different chitin components, which, in turn, affected the DD [25,47]. In future studies, higher concentrations of NaOH (60%, 65%, or 70%) could be used, and more protein and ash could be removed during chitin extraction.

Chitosan with different degrees of deacetylation has different applications in many fields. Chitosan with a degree of deacetylation above 85% to 95% can be called chitosan with a high degree of deacetylation. Chitosan with a high degree of deacetylation has been widely used in the food industry [48] because of its high free amino content, which plays a good role in the antibacterial [49,50], fresh-keeping [51,52], and antiseptic [53] areas. Chitosan with a degree of deacetylation of 55% to 70% is medium and low degree of deacetylation chitosan. This kind of chitosan has unique applications in the fields of tissue engineering [54], biomedicine [48], and environmental protection [55]. Chitosan with different degrees of deacetylation also has different functional properties. By further exploring the method of the deacetylation degree of chitosan, it is believed that it can provide help and reference for expanding its wider application.

## 5. Conclusions

In this study, chitin was isolated from BSF by using biological treatment and analyzed by FTIR spectroscopy. The FTIR identification results showed that the obtained chitins were all type α. The chitin yields of BSF pupae and adults were 59.9% and 47.31%, respectively. A notable detail is that, in the obtained chitin, the DM rate reached over 90%, whereas those of the residual protein content were 66.67% and 53.37%, and the DDs were 18.52% and 37.38%. In short, *A. pasteurianus* AS1.41 had a better DM effect on BSF, and *B. subtilis* S4 had a certain DP ability on BSF, but the effect was average. The study also found that raw materials at different developmental stages may affect chitin production. In addition, for BSF, a higher concentration of NaOH was more favorable for deacetylation. Finally, the co-fermentation of *B. subtilis* S4 and *A. pasteurianus* AS1.41 had high fermentation efficiency and good synergy. It had the advantages of no need to re-sterilize, no need to replace the medium, a short fermentation time, etc., and reduced waste water discharge and energy consumption. Therefore, the biological fermentation described in this paper is an environmentally friendly and low-cost method.

## Figures and Tables

**Table 1:** Deproteinization rate, demineralization rate, chitin yield, and chitosan deacetylation degree of different treatments.

Method	Samples	DP (%)	DM (%)	CY (%)	DD (%)
Biological	Puparium	33.33 ± 2.58 ^d^	94.92 ± 0.87 ^a^	59.90 ± 0.52 ^a^	18.52 ± 7.01 ^b^
	Adult	46.63 ± 8.39 ^c^	90.93 ± 0.31 ^a^	47.31 ± 0.40 ^b^	37.38 ± 19.27 ^ab^
Chemical	Puparium	75.36 ± 2.39 ^b^	93.83 ± 0.41 ^a^	23.82 ± 0.90 ^c^	25.73 ± 3.87 ^b^
	Adult	89.11 ± 1.72 ^a^	92.52 ± 5.38 ^a^	11.99 ± 1.39 ^d^	60.23 ± 21.28 ^a^

^a–d^ Means with different superscripts within a column indicate significant difference (*p* < 0.05). DP: deproteinization; DM: demineralization; CY: chitin yield; DD: degree of deacetylation.

## Data Availability

Not applicable.
